# Brachial plexus injury after shoulder dislocation: a literature review

**DOI:** 10.1007/s10143-018-1001-x

**Published:** 2018-06-30

**Authors:** Olga Gutkowska, Jacek Martynkiewicz, Maciej Urban, Jerzy Gosk

**Affiliations:** Department of Traumatology, Clinical Department of Traumatology and Hand Surgery, Wroclaw Medical University, ul. Borowska 213, 50-556 Wroclaw, Poland

**Keywords:** Brachial plexus injury, Infraclavicular brachial plexus, Nerve injury, Shoulder dislocation, Glenohumeral dislocation, Terrible triad of the shoulder

## Abstract

Brachial plexus injuries are among the rarest but at the same time the most severe complications of shoulder dislocation. The symptoms range from transient weakening or tingling sensation of the upper limb to total permanent paralysis of the limb associated with chronic pain and disability. Conflicting opinions exist as to whether these injuries should be treated operatively and if so when surgery should be performed. In this review, available literature dedicated to neurological complications of shoulder dislocation has been analysed and management algorithm has been proposed. Neurological complications were found in 5.4–55% of all dislocations, with the two most commonly affected patient groups being elderly women sustaining dislocation as a result of a simple fall and young men after high-energy injuries, often multitrauma victims. Infraclavicular part of the brachial plexus was most often affected. Neurapraxia or axonotmesis predominated, and complete nerve disruption was observed in less than 3% of the patients. Shoulder dislocation caused injury to multiple nerves more often than mononeuropathies. The axillary nerve was most commonly affected, both as a single nerve and in combination with other nerves. Older patient age, higher energy of the initial trauma and longer period from dislocation to its reduction have been postulated as risk factors. Brachial plexus injury resolved spontaneously in the majority of the patients. Operative treatment was required in 13–18% of the patients in different studies. Patients with suspected neurological complications require systematic control. Surgery should be performed within 3–6 months from the injury when no signs of recovery are present.

## Introduction

Traumatic anterior shoulder dislocation is the most common major joint dislocation with the incidence estimated at 2% of the population during lifetime [1, 2]. It is connected with a high number of associated injuries, resulting both from the mechanism of the initial trauma and reduction techniques [3, 4]. These injuries can affect sole or multiple tendinous, neural and vascular structures around the shoulder joint. Neurological complications resulting from shoulder dislocation include single nerve injuries, as well as more complex brachial plexus injuries (BPIs) and can cause a wide scale of disability, ranging from transient weakening of the upper limb and tingling sensation to total permanent paralysis of the limb associated with chronic pain and secondary deformities causing psychological distress.

The purpose of this work is to evaluate the incidence of neurological injuries in patients who suffered shoulder dislocation, determine which nerve(s) are affected most often and what the mechanism and severity of nerve injuries are, what type of patients are most susceptible to neurological complications and with what other injuries can nerve injury coexist. We also aim to determine how long the recovery of limb function lasts in patients treated conservatively and operatively, what percentage of patients require operative treatment and what the optimal time frame for surgical intervention is. On the basis of literature data, we aim to create a management algorithm for patients with suspected neurological injury after shoulder dislocation.

## Materials and methods

A review of studies dedicated to neurological complications of traumatic anterior shoulder dislocation was performed. Search of keywords “glenohumeral dislocation”, “shoulder dislocation”, “brachial plexus injury”, “nerve injury”, “axillary nerve injury”, “neurovascular injury”, “infraclavicular lesions”, “unhappy triad of the shoulder” and “terrible triad of the shoulder” in PubMed, MEDLINE, Scopus and Google Scholar databases from their inceptions to 28 February 2018 was conducted. Articles written in all languages, including case reports presenting single or combined neurological complications of shoulder dislocation, were included. Articles failing to report the cause of brachial plexus lesion, abstracts and duplicates were excluded. The summary of the analysed literature has been presented in Tables 1 and 2.

**Table 1:** Literature source file—articles reporting neurological complications of shoulder dislocation

Article	Number of patients	Mean patient age (age range)	Sex male/female	Side right/left	Cause of initial trauma	Associated injuries	Nerve injury	Operation
Robinson et al. [5]	492 out of 3633 (13.5%)	All patients 47.6 (13–104) BPI 42.5 BPI + GTF/RCT 57.5 BPI + GTF 56.3 BPI + RCT 63.0 All patients with nerve injury 51.5 years	All 1.6:1 BPI 2:1 BPI + GTF/RCT 1:1 BPI + GTF 0.9:1 BPI + RCT 1.2:1 All patients with nerve injury 1.3:1	All patients 54.2%:45.8%	Order: BPI–BPI + GTF/RCT–BPI + GTF–BPI + RCT–all–all with nerve injury Fall = 40.5%; 68.4%; 68%; 69.7%; 49.8%; 56.5% Fall from height = 11%; 12.1%; 13.1%; 9.2%; 10.3%; 11.6% Sports injury = 42.4%; 7.1%; 8.3%: 3.9%; 34%; 22.2% MVA = 4.8%; 6.4%; 5.8%; 7.9%; 3.6%; 5.7% Other = 1.4%; 6%; 4.9%; 9.2%; 2.4%; 4.1%	BPI + RCT/GTF 282 pts (7.8%) BPI + GTF 206 pts (5.7%) BPI + RCT 76 pts (2.1%)	BPI alone 210 pts (5.8%) BPI alone–BPI + GTF–BPI + RCT–all Axillary = 73.8%; 62.6%; 59.2%; 66.9% Ulnar = 10.5%; 11.2%; 9.2%; 10.6% Radial = 1.4%; 2.4%; 1.3%; 1.8% MSC = 1%; 1%; 2.6%; 1.2% Median = 3.8%; 1.9%; 2.6%; 2.8% Multiple = 9.5%; 20.9%; 25%; 16.7%	3 patients (tendon transfers)
Travlos et al. [6]	28	42.6 (17–82)	19:9	–	Minor fall 9; major fall 6; MVA 7; Direct blow 3; Other 3	GTF (2), clavicle frx (1), scapula frx (3), clavicle + scapula frx (1)	Infraclavicular + supraclavicular 8; diffuse infraclavicular 7; axillary 7; axillary + lateral cord 2; posterior + medial cord 1; medial cord 3	5 operated-on: 4 neurolysis; 3 grafts (axillary nerve)
Wehbe et al. [7]	33 (10 GHD)	30 (13–70)	26:7	15:18	MVA 19; sports accident 2; occupational injury 2; fall 6; automobile crash 2; positional anomaly 1; direct blow 1; extreme abduction 9; anterior GHD 10; unclear 13	Present 23: ACD 3; GTF 3; fractures: scapula 5; humerus 2; forearm 2; rib 3; clavicle 3	Axillary only 16 Axillary + suprascapular 5 Axillary + infraclavicular BPI 11 Axillary + supraclavicular BPI 1	All (20 nerve grafting; 13 neurolysis)
Liveson [8]	11	55 (28–76)	8:3	–	Fall 6; motorcycle accident 1; traction 2; recurrent GHD 1; blunt blow 1	3 factures non-specified	Axillary only 3; MSC 1 Axillary + radial + MSC + median 1 Medial cord (+posterior) 1 Posterior cord 1 Brachial plexus + suprascapular 1 Axillary + MSC 2 Axillary + branch to triceps 1 (axillary 10; MSC 5)	No
Kosiyatrakul et al. [9]	14 (15 shoulders)	47.8 (27–83)	9:5	7:6:1B	Fall 5; fall from height 1; car accident 3; motor accident 3; skiing accident 1; lifting 1	GTF 6	3 suprascapular nerve Recovery 20 months (thenar, interossei worst recovery)	3 patients (tendon transfers)
Toolanen et al. [10]	65 pts (36 nerve injuries)	64 (41–90)	36:29	39:26	–	RCT 24 (terrible triad 12)	Axillary only 30; axillary + other 5; radial ( +median + ulnar) 1	No
deLaat et al. [11]	101 (44 GHD) 14 nerve lesions	53 for GHD patients	30:71	47:54	Fall 87; sports accident 14	In GHD group: GTF 7; RCT 3	Axillary only 8; axillary + other 18; axillary 37 pts; radial 22 pts MSC 19 pts; ulnar 8 pts	No
Yeap et al. [12]	11 (out of 115 pts; 80 GHD)	35.7	8:3	–	7 fall; 2 MVA; 2 spontaneous	–	Axillary only 5, ulnar 2, median 1, diffuse BPI 1; axillary + radial 1; axillary + radial + ulnar 1	No
Pasila et al. [13]	50 (out of 238)	Most pts > 50 years	All 133:105; Complicated 34:29	–	Fall 22; fall from height 7; twist 10	RCT 28	21 axillary; 29 multiple	No
Pasila et al. [14]	44 (out of 226)	Most > 50 years	All 126:100	–	Fall 92; fall from height 15; torsion 24	26 RCT; terrible triad 6	19 axillary (6 persistent), 25 multiple (9 persistent)	No
Visser et al. [15]	37 (out of 77)	52.3 (16–94)	All 38:39 BPI 15:22	42:35	–	GTF 12 (BPI + GTF 10) RCT 7	Axillary 32; radial 5; MSC 9; median 3, ulnar 6; complete 1, single nerve injury 51%	No
Visser et al. [16]	215 pts 74 GHD 133 nerve injuries	64.2 (12–94)	58:157	–	–	–	Nerve injury in 50% of GHDs (37) Axillary – 53%; Radial-24%; MSC 24%; median 12%; ulnar 7% 30% single nerve injury; posterior cord 38%	No
Payne et al. [17]	48 (2.6% out of 1844)	45.5 (15–80) Terrible triad = 4/5 pts > 57 years of age	33:15 4:1 (triad)	3:2 (triad)	Fall 17; MVA 14; lifting 7	Unhappy triad 5 pts (10.4%); 2× axillary; 3× multiple nerve injury	Total BPI-20 pts; Mononeuropathy 17 pts (axillary 47%, MSC 6%) multiple nerves 11 pts (axillary + MSC 45%; axillary + suprascap. 36%) Triad: 2× axillary; 2× axillary + suprasc.; 2× axillary + MSC	No
Hems and Mahmood [18]	101 pts 55 GHD	Whole group 46 (14–89); BPI 52 (16–86)	62:39 32:23	–	Fall 37, fall from height 6, MVA 5, skiing 3, assault 2, fall from a horse 2	RCT/GTF 17 (31%) 1 false aneurysm	Complete 14; axillary 37; ulnar 39; median 35; radial 28; MSC 28; axillary rupture 3.6% (2 pts) median, ulnar worst recovery	8 operated (15%)
Gutkowska et al. [19]	73	Mean 50	58:15	40:33	Fall 37, fall from height 21, MVA 7, other 8	GTF 22, RCT 7, HF 4	Axillary 54, ulnar 51, median 48, radial 45, MCS 21	All operated
Strafun et al. [20]	25 (20.8% of 120 GHDs)	51.52 ± 12.97 (27–66)	18:7	13:12	–	17 RCT (16 operated-on) 8 GTF	Axillary nerve 15 Diffuse BPI 10	Operated: axillary 2; BP neurolysis 4
Fox et al. [21]	22	Mean 53	19:3	–	–	13 RCT (7 oper.) 9 GTF (7 oper.)	Mononeuropathy 4, axillary 20, MSC 6 (cords: posterior 10; medial 8; lateral 7)	All BPIs (grafting 5)
Atef et al. [22]	240	All 35.2 (20–60) only Axillary 46.3 Axil. + GTF 32.8 Axil. + RCT 53.9	176:64	216 domin:24 non-domin.	Fall 43.3%; fall from height 23.3%; trauma 20.8%, MVA 12.5% (incl. all axillary + GTF); triad–fall on outstretched hand	Axillary + RCT 6.25% (15 pts) Axillary + GTF 6.25%	Only axillary Axillary 38 pts (15.6%) Axillary alone 3.33% (8 pts) Triad age > 48 years in all pts	No
Perron et al. [23]	24 (out of 190)	34.3 for all pts	78%:22% (all pts)	48%:52% (all pts)	–	10 GTF	All axillary 75% went off after reduction	No
Gumina and Postacchini [24]	545 GHD	Elderly	–	–	–	RCT 61%	Axillary 9.3% Spontan. recovery in 3–12 months	No
Vermeiren et al. [25]	154 GHD (4 BPIs)	–	–	–	–	–	Axillary 1 Radial + ulnar 3	No
Saragaglia et al. [26]	233 GHD	–	–	–	–	–	10 (6 axillary, 4 combined)	No
teSlaa et al. [27]	105 pts 107 shoulders	42 Whole group 39	–	55:52	34% sport; 28% home	–	22 nerve injuries Axillary 13	No
Lill et al. [28]	175 GHD	–	–	–	–	–	6 nerves: axillary 4, radial 2	No
Neviaser et al. [29]	31	Mean 57.5	22:9	–	–	RCT 100%	Axillary 4	All RCTs operated
Sturm and Perry [30]	59 (6 GHD)	35.5 (2–84)	45:14	23:33:3B	MVA 53%	–	Complete BPI 8	No
Leffert and Seddon [31]	31 pts (17 GHD)	Median age 27	27:4	–	Fall 7; fall from vehicle 12, struck by car 2, MVA 4, blow 3; hyperabduction 3	12 GTF; 1 scapula fracture	Diffuse BPI 14	4 operated
Bumbasirevic et al. [32]	18	(17 > 40 years)	–	–	–	–	50% nerve injury: axillary 38,3%; MSC 22%	No

*BPI* brachial plexus injury, *GTF* fracture of the greater tuberosity of humerus, *RCT* rotator cuff tear, *MVA* motor vehicle accident, *pts* patients, *MSC* musculocutaneous nerve, *frx* fracture, *GHD* glenohumeral dislocation, *ACD* acromioclavicular dislocation, *HF* humeral fracture, , *suprascap*. suprascapular, *Axil.* axillary

**Table 2:** Literature source file—case reports

Article	Sex	Side	Cause of injury	Associated injuries	Nerve injury	Age	Operative treatment
Dhar [33]	F	R	MVA	–	Diffuse	32	No
Jerosch et al. [34]	M	–	–	–	MSC	47	–
Saab [35]	F	R	Fall from a horse	–	Diffuse	49	No
Koulali-Idrissi et al. [36]	M	R	Fall	GTF	Total BPI	52	No
Volpin et al. [37]	F	R	Fall from stairs	–	Total	62	Recovery in 9 months
Volpin et al. [37]	F	R	Fall from stairs	–	Total (ulnar nerve slowest to recover)	52	Recovery in 12 months
Ameh and Crane [38]	F	R	Fall	no	Radial + ulnar	57	No
Chillemi et al. [39]	M	L	MVA	GTF	Posterior cord	27	No
Sinha et al. [40]	M	R	Fall	–	Posterior + medial cord	71	No
Shears et al. [41]	M	L	Fall	GTF	Posterior + medial cord	32	No
Rathore et al. [42]	F	R	Fall from stairs	Proximal humerus fracture (oper.)	Median + radial	53	Good recovery in 3 months

This research received approval from Local Bioethics Committee of Wroclaw Medical University and was approved by the institution at which it was carried out.

## Incidence

The earliest known description of brachial plexus lesion as a result of shoulder dislocation comes from 1910 [43]. According to historical publications dating back to 1930s–1950s, injury to the axillary nerve was found in 5–60% of patients after shoulder dislocation [3, 44–46]. The incidence of neurological complications is similar in the current literature and varies from 5.4 to 55%, being more common in primary than in recurrent dislocations (10 and 2%, respectively, according to McLaughlin and McLellan) [5, 10, 11, 13–16, 24, 29, 46–52]. In the largest prospective study conducted on 3633 patients who suffered shoulder dislocation, the incidence of neurological deficit was 13.5% [5]. Brachial plexus injury was found in 1.2% of multitrauma patients in the study by Midha et al., with shoulder dislocation being responsible for 7% of closed BPIs [53]. Males predominated in all large patient groups (M/F from 1.3:1 in the study by Robinson et al. to 6.3:1 among the patients studied by Fox et al.) (Table 1) [5, 18–22].

BPI after shoulder dislocation is most commonly observed in two patient groups. The first one comprises patients in whom dislocation is a result of high-energy forces (usually motor vehicle accident, rarer fall from a height or sports injury). In those patients, often being multitrauma victims, multiple other injuries coexist, including fractures of the shoulder girdle, proximal humerus and the first rib, which constitute separate possible causes of injury to the brachial plexus. In the analysed literature, high-energy injury was the cause of shoulder dislocation complicated with BPI in 18–71% of the patients in different studies [5, 6, 8, 9, 12, 18, 19, 22, 51]. In four studies analysing the largest patient groups, high-energy injury was responsible for 36–43% of the cases [5, 18, 19, 22].

The other group consists of patients who sustain shoulder dislocation as a result of a simple fall. The mean patient age in this group is higher (usually over 50 years), there are typically no accompanying fractures (fracture of the greater tuberosity of humerus (GTF) can be present) and nerve injury more often has transient character [5, 19, 22]*.* Analysis of the literature revealed simple fall to be the most common cause of BPI after shoulder dislocation (18–86% of the patients in different studies), including 43–67% in the four studies on the largest patient groups [5, 18, 19, 22].

The mean age of the patients in the analysed groups varied from 30 years (Wehbe et al.) to 67 years (Stenning et al.) and in 64% of the groups it was above 50 years (Table 1) [7, 54].

## Mechanism of nerve injury

Shoulder dislocation can cause damage to the neighbouring neural structures in several different mechanisms. Historical data reported two possible mechanisms of injury to the axillary nerve during shoulder dislocation. McGregor postulated that the nerve was damaged due to being crushed between the humeral head and the axillary border of the scapula [55]. Most authors, however, believed that the lesion resulted from traction injury, raising rapid recovery and predominance of motor over sensory injury as arguments to support this thesis [56, 57]. Stevens provided the first description of this injury mechanism in 1934, stating that axillary nerve is stretched across the humeral head in the abducted and externally rotated position of the arm [57].

Two other confirmed mechanisms of nerve injury after shoulder dislocation are connected with accompanying damage to vascular structures. In such cases, symptoms of nerve injury occur tardily [58, 59]. Formation of an expanding haematoma in the axillary region, close to the neural structures, causes compression and stretching of neural elements in the initial days and weeks after trauma and leads to scarring of the surrounding tissues and formation of adhesions further compressing elements of the brachial plexus [6–8, 38, 41, 58, 60, 61]. Rarer, vascular injury may lead to formation of pseudoaneurysm or false aneurysm of the axillary artery which causes delayed compression of neural structures, characterised by unaltered limb function immediately after dislocation, with its gradual deterioration over the following days to months [18, 62, 63].

The least information can be found about the fourth mechanism—injury to vasa nervorum causing ischaemia of peripheral nerves resulting in impairment of their function [54]. Insufficiency of vasa nervorum may result from both direct injury to the arterioles, which are especially susceptible to compression, and from injury to a major vessel [54]. Prolonged ischaemia leads to infarction of nerve which is equal to neurotmesis [54].

In some cases, the injury to neurovascular structures may be caused by abrupt or brutal reduction manoeuvre. Certain reduction techniques are more likely to cause such complications, including Hippocrates method, in which violent traction applied to outstretched arm combined with compression in the axillary region by the operator’s heel may result in injury to neurovascular structures [4, 42, 59]. It is, therefore, essential to assess and document the neurological status of the limb, both before and after reduction attempt [51].

## Affection of particular nerves

Out of all BPIs, those affecting its infraclavicular part constitute only 25% [60, 64]. However, injuries resulting from shoulder dislocation affect predominantly the infraclavicular part of the plexus at the level of cords and nerves, often extending up to retropectoralis minor space [11, 65, 66]. It may be explained by the fact that trauma in abduction causes primarily injury to the lower part of the brachial plexus [64]. Position of the limb during dislocation has been reported to influence the site of nerve injury. Major traction forces act upon the medial cord when the elbow and wrist are extended, the medial and posterior cords when the elbow is flexed and all cords when the arm is in 90° abduction and full extension [35, 41, 60]. In the position of extreme abduction and internal rotation of the arm, which is observed during motorcycle accidents and causes opening of the scapulohumeral angle with wedging of the infraclavicular brachial plexus, injury to the nerves located closest to the shoulder joint, especially axillary, musculocutaneous and radial nerves, is most often found [7]. Pulling down by the limb in internal rotation causes injury to the motor branch of the axillary nerve, which winds around surgical neck of the humerus and leaves the more distally located sensory branch intact [7].

Shoulder dislocation most commonly causes injury to the axillary nerve, both when single nerve injury is considered and in combination with other nerves [5, 11, 16, 18, 42]*.* Axillary nerve was found to be affected in all patients with neurologic deficit after shoulder dislocation (100%) in several studies [6, 7, 22, 23, 51, 67–70], and in all analysed studies but two (Hems and Mahmood, Stenning et al.), it occupied the first place among injuries to the long nerves of the brachial plexus [18, 54]*.*

BPI manifested as mononeuropathy in 18.2% (Fox et al.) to 90.5% (Robinson et al.) of the cases [5, 7, 8, 10–12, 15–17, 19, 21, 22]. In general, multiple nerve injuries were found more often than injury to a single nerve [7, 8, 12, 16, 19, 21, 22]*.* In the study by Robinson et al., injury to the axillary nerve alone was more common in young individuals and as a result of high-energy trauma, while complex neurological deficits were associated with older age, female sex and low-energy fall [5]. Another study confirmed that single nerve injury is more likely to be a result of high-energy trauma in a younger patient, while multiple nerve injury would be a result of low-energy fall in an elderly patient [19]. In the previously cited study by Robinson et al., percentage of multiple nerve lesions was significantly higher in the group with other associated injuries (rotator cuff tear (RCT)/GTF) [5].

The incidence of injuries to other nerves of the brachial plexus varied in different studies. In two studies comprising the largest numbers of patients, particular nerves were affected in the descending order of frequency: axillary, ulnar, median, radial and musculocutaneous nerve [5, 19]. By contrast, in two other studies on relatively large patient groups, musculocutaneous nerve occupied the second position [15, 21].

In the studies analysing BPI after shoulder dislocation with reference to injury to particular cords, posterior cord was the most common to be injured [16, 21, 60].

Total brachial plexus palsy (affection of all five long nerves of the brachial plexus) was observed in 2.7% [15] to 41.7% [17] of all nerve injuries, and in the studies by Robinson et al. and Gutkowska et al., it was associated with the presence of GTF [5, 18, 19, 30].

Shoulder dislocation can cause injury not only to the long nerves but also the short branches of the brachial plexus. Out of the short nerves of the brachial plexus, suprascapular nerve injury has been reported in the largest number of studies [7–9, 17, 71, 72]. Its distance from the posterior glenoid rim has been reported to be averagely 1.8 cm and its relative fixity at the scapular notch makes this nerve susceptible to traction injury [56, 72–75]. The symptoms of suprascapular nerve injury are often vague and unspecific, manifesting as pain and muscle weakness [74]. The clinical images of axillary and suprascapular nerve injuries overlap and are difficult to differentiate without nerve conduction studies. Injury to these two nerves can also coexist, which leads to severe impairment of arm movement [7, 17, 71]. The clinical picture may also resemble RCT and even shoulder instability [74]. Clinical examination and electromyography (EMG) of the supraspinatus and infraspinatus muscles should be performed. The treatment is nonoperative in the majority of the cases but spontaneous recovery may take more than 1 year [72, 74]. However, in some cases, this injury may require operative treatment (neurolysis) in order to relieve pain, improve spinati muscles function and prevent their atrophy [71, 74]. Kline et al. observed that in cases of combined axillary and suprascapular nerve injury, suprascapular nerve function improved spontaneously in the majority of the cases, while axillary nerve more often required operative intervention [71].

## Severity of lesions

Nerve injury complicating shoulder dislocation most often is neurapraxia or axonotmesis according to Seddon or first to fourth degree injury according to Sunderland [33, 42, 54, 56, 67, 75, 76]. Complete disruption of nerve continuity or its avulsion is very rare due to the fact that traction is exerted at a point relatively distant to the point of exit of nerve roots from the spinal cord and the plexus is mobile and extensible at this level, as well as thanks to the relatively low energy of injury being in most cases simple fall [6, 33, 35].

In the analysed literature, complete nerve disruption was rare and observed only for the axillary nerve. It occurred in 2.4% of the patients in the study by Hems and Mahmood and in 2.7% of the patients analysed by Gutkowska et al. (in all cases as a result of high-energy trauma) [18, 19]. In cases of terrible triad, neurotmesis requiring repair with nerve grafting was more frequent and occurred in 22.7% of the patients in the study by Fox et al. and in 29% of the patients studied by Rovesta et al. [21, 51].

## Risk factors for neurological complications

Elevated risk of neurological injury after shoulder dislocation is associated with a number of variables. The most important of them is higher patient age [5, 10, 12, 14, 77]. Several studies confirmed that mean age of the patients who sustained isolated shoulder dislocation was lower than those who suffered neurological complications [18, 22, 23, 27, 28]. Visser et al. found the probability of neural injury to increase with a factor of 1.3 per every 10-year period [15]. Due to predominance of men in the studied patient groups, male sex can also be considered a risk factor. However, in the comprehensive study by Robinson et al., demographic features of the group of patients with neurological deficit alone did not differ from the general population of dislocators [5].

Conflicting opinions can be found in the literature regarding the influence of the energy of the initial trauma causing dislocation on the risk of BPI. High-energy trauma has been postulated to be connected with an elevated risk by Pasila et al. and Yeap et al., while Robinson et al. found more neurological complications in patients who suffered low-energy injury [5, 12, 13].

Longer time period between dislocation and its reduction has been associated with higher risk of neural complications [12, 14, 19]. More neural complications have also been observed after first time than after recurrent shoulder dislocations [14, 49].

As far as associated injuries are concerned, according to Robinson et al., the likelihood of neurological deficit is significantly higher for patients with coexisting RCT or GTF (RR 1.9) [5]. In the study by Visser et al., the presence of GTF doubled the incidence of nerve injury [15].

Higher incidence of nerve injuries was also connected with the presence of coexisting haematoma, with the adverse effect noticeable both in terms of severity of injury and the number of nerves involved [11, 15].

## Accompanying injuries

In the study by Robinson et al., in 5.8% of the patients, neurological deficit was the only complication of shoulder dislocation, while in 7.8%, it was found together with RCT (2.1%) or GTF (5.7%) [5]. In another study on 240 patients, only 3.3% of them suffered isolated neurological deficit, while in 6.25% of the cases, it was associated with RCT or GTF [22]. Hems and Mahmood found GTF/RCT in 31% of their patients treated for injury to the infraclavicular part of the brachial plexus [18]. Patients diagnosed with neurological deficit and RCT were characterised by higher mean age in comparison to the whole patient group, while coincidence of GTF and nerve injury was generally found in younger patients (Table 3).

**Table 3:** Mean age of the patients with and without accompanying injuries

Article	Whole patient group	BPI+GTF	BPI+RCT
Robinson et al. [5]	51.5 years	56.3 years	63.0 years
Gutkowska et al. [78]	50 years and 1 month	48 years and 8 months	54 years and 8 months
Atef et al. [22]	35.2 years	32.8 years	53.9 years

### Rotator cuff tear/fracture of the greater tuberosity of humerus

Association of BPI and RCT after shoulder dislocation was first described by Gonzales and Lopez in 1991 [79] and is known under the term “unhappy triad” [80] or “terrible triad” [81] of the shoulder [79–80]. This complex injury is found more often in patients over 50 years of age and usually coexists with injury to the axillary nerve alone [9, 10, 15, 29, 66, 70, 82]. The incidence of terrible triad varied between 2 and 18% in the analysed literature [5, 10, 11, 13–15, 17, 19, 20, 22] (Table 4). Inability to initiate abduction and weakening of external rotation of the arm should raise the suspicion of a complicated dislocation [29, 70, 79, 81, 83]. Differentiation between RCT and nerve injury as causes of shoulder disability after dislocation can be difficult based on clinical examination alone [67]. According to some authors, axillary nerve injury does not cause complete absence of shoulder abduction so this symptom indicates the presence of RCT [18, 51]. Out of the two, RCT is a more common cause of upper limb weakening and pain in older patients due to degeneration of collagen fibres composing tendons, which progresses with age [5, 51, 70]. Displaced GTF is a functional equivalent of rotator cuff discontinuity [51, 79]. Association of GTF and nerve injury was found in 5.7–32% of the patients in the analysed literature [5–7, 11, 15, 19, 22]*.* Robinson et al. suggest that “in the absence of GTF on postreduction radiograph, it is advisable to image the rotator cuff in patients with more complex neurological deficit” [5]. To differentiate between RCT and nerve injury, magnetic resonance imaging (MRI), computed tomography (CT) arthrography or ultrasound should be performed as soon as possible to confirm RCT and avoid unnecessary intervention on the brachial plexus [67, 83]. However, even when RCT has been diagnosed, EMG testing for nerve injury should be conducted, as the two injuries often mask each other [70, 79, 80]. RCT requires early operative repair, especially in younger patients in order to improve functional recovery and avoid muscle atrophy, while the approach to nerve injury should be conservative in the initial phase [5, 10, 12, 17, 18, 20, 21, 41, 51, 66, 70, 79–80, 83, 84, 86, 87]. However, if adequate RCT reconstruction does not cause the limb movement to improve, nerve function should be reassessed and operative treatment considered [17, 18]. According to Strafun et al., if in preoperative EMG examination more than 30% of axillary nerve conduction is preserved, the patient should be operated-on for RCT and the treatment of neural injury should be conservative, but if conduction is less than 30%, early surgical exploration of axillary nerve is advocated [20]. Simonich et al. concluded that the final functional result of the affected limb is more dependent on nerve recovery than on complete RCT repair [70].

**Table 4:** Summary of literature on unhappy triad of the shoulder

Author, year	Age	Sex	Side	Mechanism	Injury	Treatment	Outcome
Goubier et al., 2003 [83]	27	M	L	Motorcycle accident	Supraspinatus, infraspinatus Retroclavicular BP palsy; GTF	RCT–oper. BPI–conserv.	Full recovery of diffuse BPI in 12 months
Gonzales and Lopez, 1991 [79]	57	F	R	Assaulted	Full thickness RCT Axillary, MSC	RCT–oper. BPI–conserv.	Resolution of symptoms in 3 months
Gonzales and Lopez, 1991 [79]	66	M	L	Struck by a car	GTF, medial cord (complete), lateral cord (incomplete)	Conservative	Lateral cord—complete recovery in 2 years; medial cord—no recovery
Groh and Rockwood, 1995 [81]	57	F	R	Fall	Full thickness RTC Axillary n., incomplete	Conservative	Complete recovery in 6 months
Groh and Rockwood, 1995 [81]	41	M	R	Motorcycle accident	Full thickness RTC Axillary n., incomplete	RCT–oper. BPI–conserv.	Complete recovery in 3 months
Güven et al. 1994 [80]	53	M	L	Struck by a car	RCT; total BPI	RCT–oper. BPI–conserv.	Spontan. recovery in 3 months
Miller et al., 2012 [84]	42	M	R	Fall from 10 m	RCT; axillary nerve	RCT – oper. BPI–conserv.	Recovery in 6 months
Simonich and Wright, 2003 [70]	Mean 57 (37–79)	5 M + 1 F	3R 3 L	–	Full thickness RTC 5 axillary; 1 axillary + 1 SSC	RCT–oper. 1 axillary–oper. SSC–oper.	5/6 BPI–recovery in 12 months; 1 axillary (oper. after 10 months)-persistent palsy
Takase et al., 2014 [66]	61	F	R	Fall	RTC + axillary n. + glenoid rim frx (terrible tetrad)	RCT–oper.	Nerve recovery, 3 months
Mehta and Kottamasu, 1989 [85]	53	M	R + L	Fall	RCT (R) + diffuse BPI (R)	Conservative	Gradual recovery
Brown et al., 2000 [67]	Mean 65	6 M + 9 F	–	–	12 axillary + 4SSC + 1MSC	13 RCT–oper.	Nerve recovery: 8 complete 7 incomplete
Rovesta et al., 2015 [51]	47 (21–72)	24 pts 21 M + 3 F	–	17 high-energy 7 low-energy	Axillary, all (18, as a single nerve)	7 conservative 10 neurolysis 7 grafting	Nerve recovery: 8 good 10 medium 6 bad
Prudnikov 1994 [69]	–	22 pts (20 GHD)	–	–	Axillary, all	All RCT–oper.	4 persistent palsy
Johnson and Bayley, 1982 [82]	51 (31–76)	12 pts 9 M + 3 F	–	–	Axillary, all 4 axillary + other nerve	5 RCT, 7 GTF 9–oper.	2 good 3 fair 4 poor

*RCT* rotator cuff tear, *GTF* fracture of the greater tuberosity of humerus, *oper*. operative, *pts* patients, *GHD* glenohumeral dislocation, *Spontan*. spontaneous

### Neurovascular injury

A rare but devastating sequelae of shoulder dislocation is complex neurovascular injury with trauma to both brachial plexus and axillary or rarer subclavian artery [37, 59, 61]. Vascular injury is observed in up to 25% of infraclavicular lesions [60]. In the analysed literature, 29 cases of neurovascular injury complicating shoulder dislocation have been identified, out of which 75% occurred in patients older than 60 years (Table 5). This injury is more common in elderly patients, whose arteries are atherosclerotic, less elastic and therefore more susceptible to tear as a result of forced traction during dislocation or reduction manoeuvres [18, 61–63, 88–91]. Axillary artery is injured in its third portion (between the inferior borders of pectoralis minor and teres minor muscles) in 90% of the cases [88]. In the position of abduction and external rotation of the arm, the artery becomes tense [89]. The mechanism of injury is complex: the artery is suddenly pulled and stretched over the edge of pectoralis minor muscle which acts as a fulcrum, in case of recurrent dislocation or arthritic changes of the shoulder joint it can be torn by adhesions existing between its walls and the surrounding tissues, and the dislocated humeral head exerts pressure on the artery [18, 61, 88]. At the level of the axilla, brachial plexus and axillary artery are invested by a common connective tissue sheath [54]. Thus, even minimal swelling within the sheath can cause compression on plexus elements [54]. Expanding haematoma or rarer pseudoaneurysm contributes to deterioration of neural function of the limb [11, 15, 18, 31, 38, 59, 62, 63, 89]. Moreover, occlusion of axillary artery may result in nerve ischaemia [54, 59]. The onset of nerve palsy in such cases is delayed and the symptoms worsen in time [59, 62, 63]. Therefore, evidence of plexopathy or isolated neuropathy after shoulder dislocation should always raise a suspicion of coexisting arterial injury [62, 68, 93]. Similarly, the presence of vascular injury may provide information about the site and severity of nerve lesions [86]. According to Stenning et al., particularly close relationship between median nerve trunk and the axillary artery causes this nerve to be most commonly involved in periarterial fibrosis or incorporated into the wall of pseudoaneurysm [54]. In the analysed literature, arterial injury in the majority of the cases coexisted with diffuse or complete injury to the brachial plexus. Due to rich collateral circulation, the presence of palpable distal pulses does not preclude axillary artery injury [18, 59–62, 88, 91]. In case of suspected arterial injury, CT angiography or arteriography is required to confirm the diagnosis, followed by immediate surgical intervention to reconstruct the arterial defect and evacuate haematoma. Brachial plexus should be simultaneously explored, subjected to decompression and external neurolysis to relieve pressure on nerves, avoid irreversible neurological damage and promote spontaneous recovery [18, 54, 58, 59]. According to Shaw et al., the long-term outcomes in neurovascular injury depend more on nerve regeneration than arterial injury, which can be easily repaired operatively [93].

**Table 5:** Summary of literature on neurovascular injury following shoulder dislocation

Author, year	Age	Sex	Side	Mechanism	Vascular injury	Nerve injury	Recovery
Allie 2005 et al., [88]	60	M	L	Fall	Axillary artery	Below C5 level	ivr; good recovery after 6 weeks
Nikolaou et al., 2008 [89]	74	M	L	Fall	Axillary artery	Median, ulnar, radial	ivr Recovery in 12 months
Helm and Watson, 2002 [63]	68	M	L	Lifting, fall, bilateral, recurrent GHD	Axillary artery pseudoaneurysm	Total BPI	pvr after 5 months + brachial plexus neurolysis; injury persistent at 9 months
Razif and Ramalingam, 2002 [61]	25	M	L	MVA	Axillary artery	Axillary	ivr Improvement after 6 months
Mullett et al., 1998 [90]	62	M	R	Fall	Axillary artery	Diffuse	ivr Partial recovery at 9 months
Emadian 1996 [62]	83	F^b^	R	Fall	Axillary artery pseudoaneurysm	Axillary	pvr NDA
Mwipatayi et al., 2005 [91]	37	M	R	Knee-boarding	Axillary artery pseudoaneurysm	Total BPI	ivr + brachial plexus neurolysis NDA
Regauer et al., 2014 [4]	69	M	R	Hippocrates reduction	Brachial vein	Diffuse	ivr + brachial plexus neurolysis Recovery in 6 months
Murata et al., 2008 [58]	16	M	R	MVA	Axillary artery	Good	Neurolysis on the 3rd day; good result
Nash et al., 1984 [59]	76	M	L	Hippocrates reduction	Subscapular artery	Median	ivr Partial recovery after 6 months
Curley et al., 1988 [92]	17	F	L	Simple reduction of recurrent GHD	Subclavian artery	Total BPI	Ehler-Danlos syndrome arm amputation
Shaw et al., 1995 [93]	3 patients	–	–	–	Axillary artery	Diffuse	Recovery poor in 2 patients, good in 1 patient
Stenning et al., 2005 [54]	Mean 67 range 43–88	20 patients (16 GHDs)	–	Low-energy injuries	Axillary artery	Median 20, ulnar 19, radial 19, MSC 17, axillary 12	irv/pvr + brachial plexus neurolysis Bad result in 1 median, 1 axillary nerve

*M* male, *F* female, *L* left, *R* right, *BPI* brachial plexus injury, *GHD* glenohumeral dislocation, *MVA* motor vehicle accident, *NDA* no data available, *MSC* musculocutaneous nerve, *ivr* immediate vascular repair, *pvr* – postponed vascular repair

## Percentage of patients requiring operation

Infraclavicular lesions require operative treatment significantly less often than injuries to supraclavicular brachial plexus and in the majority of the cases spontaneous improvement or return of limb function can be expected after a period of observation and/or rehabilitation [11, 15, 53, 60, 64, 66]. Spontaneous recovery of injured nerves has been described in 75–100% of the patients in some studies [15, 23, 24, 26]. Still, regenerative capacity decreases with age, which leads to complete lack or only limited recovery, with residual reduction of limb mobility observed in up to 60% of the patients [14, 38, 82].

In two large studies on 819 and 1019 patients, only 17 and 14%, respectively required operative treatment for stretch/contusion to infraclavicular part of the brachial plexus [94, 95]. In another study on multitrauma victims, 17% of the patients with infraclavicular lesions as opposed to 52% with supraclavicular lesions required operative intervention [53]. Similar numbers (13–18% of patients requiring surgery for BPI after shoulder dislocation) have been reported by other authors [6, 18, 31].

## Time frame for operation

Cease of neural impulsation to an effector muscle causes denervation which in the early stage is manifested by oedema and in time leads to fatty degeneration of the muscle [96]. After 2–3 months, decrease by 50% in the fibre diameter can be observed and after another 1–2 months massive accumulation of interstitial collagen begins [64, 97]. Intramuscular fibrosis impairs muscle mechanical function and prevents intramuscular axonal regeneration, which in turn affects neuromuscular synaptogenesis via changes in expression of myogenic regulatory factors, neurotrophic factor receptors, nicotinic acetylcholine receptor and nerve cell adhesion molecule, causing reduction in the number of motor end plates [97]. Such changes can be observed as early as after 3 months from the injury [98]. After 2 years, muscle fibre disintegration can be seen and between 1 and 3 years muscle fibres are replaced with adipose and fibrous connective tissue [64].

Timing of brachial plexus surgery after shoulder dislocation remains a controversial issue, which needs balancing between allowing time for spontaneous nerve regeneration and undertaking operative intervention before denervation atrophy occurs that would render the muscles refractory to reinnervation [99]. It is generally accepted that more than 12–18 months’ interval between denervation and reinnervation causes the return of muscle function unlikely to be successful [97]. More satisfactory sensory recovery can also be expected when the time interval between injury and surgery is shorter [100].

Optimal time frame for operative intervention in closed BPIs is considered between 6 and 9 months, according to current literature [101]. It allows time for the regenerating axon to reach its target muscle before irreversible degeneration of motor end plate [87, 97, 101–103]. Most authors believe that operative intervention should be postponed until 3–6 months after the injury, because it is impossible to differentiate between neurotmesis and neurapraxia before the latter wears off [6, 7, 27, 39, 42, 51, 60, 102, 104, 105]. According to Battiston et al., the optimal interval between the injury and surgery is 5.4 months [60].

However, early surgery within 3 months from the injury is being advocated by a growing number of authors. Early operative treatment has been postulated to prevent formation of perineural scar compressing healthy nerve fascicles and further worsening of symptoms [21, 60, 65, 106]. Patients with confirmed nerve discontinuity are best candidates for early operative reconstruction, which can be especially beneficial in this patient group, in which there is no chance to obtain improvement without surgical intervention. In cases of preserved nerve continuity and lack of any regenerative signs, both clinically and in EMG examination, nerve reconstruction can also be performed early, within 3 months from the initial trauma. The advantages of early surgical intervention include early reinnervation before end plate degeneration and irreversible changes in the effector muscles, alleviation of pain associated with neural injury and prevention of neuroma formation with regrowth of axons into the scar tissue [21, 60, 65, 106].

New or improved, safe and non-invasive imaging modalities have recently gained importance in diagnosis, decision-making and treatment of peripheral nerve injuries. These include magnetic resonance neurography (MRN) and high-resolution nerve sonography (frequency = 7–12 MHz or more) [107, 108]. They enhance diagnostic accuracy and help in determination of surgical feasibility and planning. The fact that these imaging techniques can provide useful information immediately after the injury, as opposed to EMG, which requires a delay before it becomes diagnostic, is an important advantage [107]. As a result, in some cases, early effective treatment can be implemented instead of following the wait-and-see strategy. When performed and interpreted by an experienced examiner, these techniques are able to adequately depict nerve disruption manifested by abrupt termination of the nerve and oedema of the surrounding tissues, massive haematomas or large neuromas, along with precise identification of the level of injury [107–111]. Large haematomas revealed in the infraclavicular region should be evacuated and brachial plexus inspected concurrently before resulting fibrosis causes compression of neural elements. Similarly, in a rare event when shoulder dislocation results in axillary nerve disruption, the nerve would benefit from an early reconstructive procedure. It is difficult to distinguish between nerve disruption and closed nerve injury that has chances for recovery on the basis of clinical examination and EMG alone [107]. In such cases, MRN helps to determine whether surgery would be beneficial [107].

## Preferred operative method

The type of surgical procedure depends on severity of injury to neural structures, time elapsed from the trauma to operation and response to electrophysiological and nerve action potentials testing. Careful pre- and intraoperative assessment of the severity and type of nerve lesion is extremely important, because unnecessary resection of a regenerating brachial plexus element or performing solely neurolysis of elements for which there is no chance for regeneration does the patient a great harm [99]. External neurolysis is sufficient in cases with nerve continuity and present regenerative nerve action potentials (NAPs) [60, 78, 112]. In such cases, the reason for impairment or loss of limb function is external scarring (fibrosis) causing compression on the elements of the brachial plexus. Microsurgical decompression performed early after trauma with the use of operating microscope or loupe magnification can lead to improvement in nerve conductivity resulting in improved limb function. Neurolysis needs to be performed in a subtle manner in order to avoid fascicular devascularisation [113]. Additional internal neurolysis is required when severe neuropathic pain accompanies or when thickened epineurium compressing nerve bundles and causing compromise of vasa nervorum is observed intraoperatively [60, 78, 112, 114, 115]. When disruption of nerve continuity is observed or regenerative NAPs are absent in a continuous nerve, grafting (usually with the use of sural nerve) should be implemented. However, according to some authors, nerve resection and grafting are not recommended during primary operative intervention, because in certain injury patterns improvement in nerve function after operation is possible only after axon regeneration (enabled by restoration of blood flow to the nerve by means of neurolysis) has been completed [77, 78, 94]. During operative exploration of the injured brachial plexus, anatomic relations of particular structures are usually altered due to the presence of a fibrous scar [94]. Coexisting injuries or status after previous surgical interventions in the axillary region (RCT repair, humeral fracture stabilisation, arterial repair) make the operation even more demanding and challenging.

After operative treatment, the patient should be followed-up for at least 2 years and preferably 5 years [87]. If more than 18 months passed between the injury and surgery, nerve repair has little chance to result in any improvement and tendon transfers, muscle transposition or arthrodesis should be considered to restore basic function of the affected limb [87, 102].

## Recovery

Infraclavicular lesions are generally considered to be milder and associated with better prognosis for recovery [94]. However, Kim et al. in their retrospective study covering more than 1000 patients with BPIs treated over a 30-year-long period found that functional loss in infraclavicular lesions was equally severe and resistant to resolve in time [95]. Moreover, they also concluded that stretch injuries have worse prognosis than sharp ones because the affected part of the nerve is longer [21, 95]. Similarly, Terzis et al. having analysed 204 cases of BPI did not confirm tendency towards more favourable outcomes in infraclavicluar lesions [116].

In the course of conservative treatment, first detectable signs of reinnervation can be seen after 1–2 months or, according to other authors, after 3–4 months and it is when initial improvement in nerve function can be expected [60, 64, 117]. These observations confirm the validity of performing serial EMGs to prognosticate and make recommendations for treatment (wait-and-see strategy versus operative intervention). Sensory recovery precedes motor recovery and constitutes a good indicator [6, 33]. Deep pressure sensation has been suggested to be the best indicator of recovery potential [6]. Evidence of early recovery may be detectable in EMG weeks to months before clinically apparent limb function improvement [87].

First signs of postoperative recovery can be expected 2 months after neurolysis or 3 months after grafting, and the regeneration process is usually complete by the 6–18th month after operation [6, 7]. The worst results regarding motor recovery have been observed for the intrinsic muscles of the hand [6, 9, 18]. This is caused by a long distance that needs to be covered by regenerating axons and the tendency towards quick atrophy of these muscles [9, 18]. Improvement in function of intrinsic muscles of the hand can be expected after a significantly longer period of time, up to 36 months [31].

### Factors influencing recovery

A number of factors influencing nerve recovery have been identified.

Wehbe et al., having analysed the results of operative treatment of 33 cases of axillary nerve injury, determined that recovery was better in patients below 25 years of age, while Battistion et al. connected higher chances for spontaneous recovery with patient age below 40 years [7, 60]. Relationship between recovery potential and patient age has also been confirmed by Visser et al. [15].

Increasing severity of nerve lesions, requiring more invasive operative treatment, correlates with poorer functional outcome [21, 60]. The best recovery of muscle strength has been observed in patients who did not require operative intervention, the results were slightly worse in patients who required neurolysis and even worse in those, who had to have nerve grafting performed [51]. Superior results obtained after neurolysis in comparison to grafting have been confirmed by other authors [7, 95]. In case of grafting, according to Wehbe et al., recovery was better when the graft length was below 6 cm [7]. The relationship between the graft length and the outcome has not been confirmed by other authors [6, 102, 114].

Another important factor influencing the recovery was time period elapsed between the initial trauma and surgery. All authors analysing this factor agreed that the outcomes were better when the operation was performed early, preferably within 6 months [7, 19, 21, 86, 114, 116].

Conflicting evidence has been found regarding the influence of injury to a single or multiple nerves on recovery. While Wehbe et al. observed better recovery in lesions of an isolated nerve, other authors found isolated lesion of axillary nerve to be associated with worst prognosis [6, 7, 19].

The results depended also on the most affected cord—they were best for lateral cord, medium for posterior cord and least favourable for medial cord, especially the ulnar nerve [95]. Inferior results and longer time required for recovery of the median and ulnar nerves have been observed by many authors [9, 18, 31, 37, 77, 78].

Some authors noticed that recovery was better when no associated lesions were present [7, 60].

## Summary

**Fig. 1:**
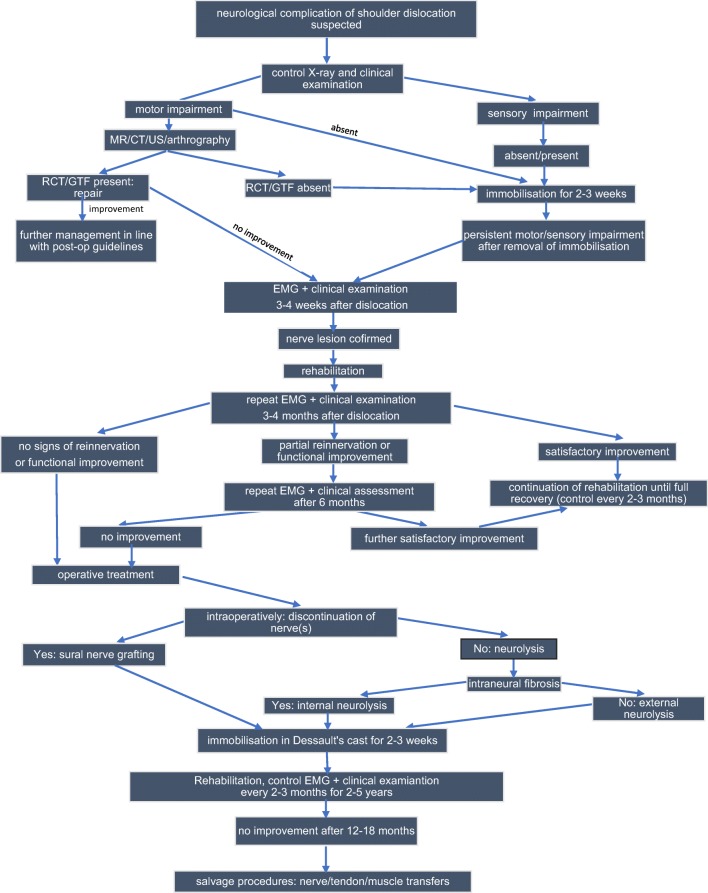
Management algorithm in patients with suspected neurological injury after shoulder dislocation

Loss of shoulder motion after dislocation, especially in older patients, is often attributed to immobilisation and stiffness, which may mask neurological injury [42]. All patients manifesting muscle weakness or altered sensation after shoulder dislocation require systematic control. The first EMG examination should be performed with a delay of at least 3 weeks because only then fibrillation potentials as a sign of denervation become evident [15, 18, 60, 64, 66, 84, 102, 117]. Nonoperative treatment is a commonly recommended approach in infraclavicular BPIs resulting from shoulder dislocation [6, 11, 18, 31, 66]. However, operative intervention should be considered when conservative approach does not bring improvement in a maximum period of 6 months. According to a growing number of authors, early surgery within 3 months from the initial trauma is especially beneficial. Recommended management algorithm in neurological complications of shoulder dislocation has been presented in Fig. 1.

Physiotherapy plays an extremely important role in the management of BPI. It prevents range of motion limitations, muscular contractures, joint stiffness, muscle atrophy, development of secondary deformities and helps supress pain. Its major goal is to maintain adequate muscle trophism during reinnervation [64, 87].

Treatment of BPI requires long-lasting cooperation between the patient, surgeon, physiotherapist and often also psychological support in order to obtain useful recovery of limb function [84]. In a satisfaction survey conducted among patients who underwent surgery to treat traumatic injury to the brachial plexus, 87% of the patients were satisfied with the outcome of operation and 83% claimed they would undergo the operation again [118].
